# A review of falls and injuries of nursing home residents presenting to the emergency department

**DOI:** 10.1093/qjmed/hcaf008

**Published:** 2025-01-12

**Authors:** Aoife O’Connor, Claire Noonan, Aoife Fallon, Sean Kennelly

**Affiliations:** Tallaght University Hospital, Dublin, Ireland; Trinity College Dublin, Dublin, Ireland; Tallaght University Hospital, Dublin, Ireland; Tallaght University Hospital, Dublin, Ireland; Trinity College Dublin, Dublin, Ireland; Tallaght University Hospital, Dublin, Ireland; Trinity College Dublin, Dublin, Ireland

## Abstract

**Background:**

Falls are frequently reported within the HSE. The Irish Longitudinal Study on Ageing (TILDA) found that 40% of over 50 s experience a fall in a two-year period, with 20% requiring hospital attendance. It has been estimated that the cost of injuries related to falls in older people will increase exponentially over the coming years. There is no national database in Ireland with statistics for nursing home (NH) residents presenting with falls to our Emergency Departments (EDs).

**Aim:**

To review the prevalence and risk factors for nursing home patients presenting to the ED with falls.

**Design:**

Retrospective chart review.

**Method:**

Retrospective review of all NH presentations to the ED of a university hospital over one year.

**Results:**

There were 519 ED presentations by NH residents over one year. 48.17% (*n* = 250) presented with a fall. One-third of ED visits presented during conventional working hours. Falls patients were more likely to be admitted when not reviewed by a GP prior to presentation. The average length of stay for falls admissions was 10.77 days (*n* = 132), versus 9.56 (*n* = 153) for admissions with no documented fall. There was no statistical difference in the falls risk medications between the groups. Patients presenting with falls were more likely to have bone protection reviewed during their stay (*P* = 0.011). Patients with falls were also more likely to use mobility aids (*P* < 0.001).

**Conclusion:**

Rapid referral to falls prevention programmes and the use of standardized care pathways for high falls risk patients are essential for future prevention and management.

## Introduction

Emergency departments (EDs) provide an extensive array of emergency care for patients from nursing homes, who require either crucial attention for life threatening illnesses or some less critical care in the hospital setting. EDs have a fundamental rule in the community in providing care and access to hospitals’ healthcare systems. While emergency care is generally of high quality, the department may not have the necessary resources for the multifaceted medical issues and needs of older people.^1^ Falls and fractures are significant public health issues, especially as the incidence increases with age. In Europe, it has been estimated that 30% of the population over 65 years of age, will fall each year, and that medical attention will be required for a fifth of these falls. Fractures will result in 10% of said falls.^2^

There are currently 576 registered nursing homes in Ireland, comprising of 32 000 residential places.^3^ The worldwide population of older people is projected to increase significantly. The population in Ireland over the age of 65 is currently 742 300 according to the most recent census.^4^ This is projected to reach 1.6 million by 2051. The change in demographics has many implications for the health service in Ireland. The economic burden of falls and fractures will increase as the population ages.

There are many factors that have been identified having an association with an increased falls risk. The proper functioning of the neuromuscular musculoskeletal and sensory systems is necessary for normal gait and the stability of posture. Proprioception and tactile, visual and vestibular input are crucial to maintenance centre of gravity. These sensory pathways may become defective or inhibited with age and disease.^5^ Impaired vision, sensory impairment and decreased strength in the lower limbs, and reduced grip strength, are all associated with falls risk.

Another major risk factor for falling is polypharmacy, also called fall risk increasing drugs (FRIDs). These include psychotropic drugs such as hypnotics, antidepressants, sedatives, antiepileptic's, antipsychotics and opioids. These medications commonly cause side effects such as delirium, sedation, in coordination, hyponatraemia. Cardiovascular drugs can cause orthostatic hypotension, and others can induce arrythmias, by increasing the QT interval.^6–8^

There is a threefold risk of falling associated with the use of a walking aid and history of falls. A history of falls may hide the underlying factors causing falls such as impaired balance.^9^ Using aids to assist with walking may also imply that these people are, in fact, active and walking and that the opposite is true of those that do not use walking aids.^10^

An understanding of the pattern of the utilization of the ED by nursing home residents, who present with falls, will have implications for planning and improving interventions to address this population's needs. There is some international evidence for innovative strategies that are safe between emergency medicine and geriatric medicine to preclude hospital admissions. Strategies for admission avoidance include rapid access clinics for the elderly and direct admissions to geriatric respite and short stay units. Currently, services such as these are not nationally available in a consistent fashion.

## Materials and methods

### Study design and setting

This study comprised of a single jurisdiction population based retrospective analysis of consecutive ED presentations of nursing home residents to the ED. The study was conducted at the ED in Tallaght University Hospital, Dublin. Data were collected by the registered Advanced Nurse Practitioner in Gerontology. It was conducted over a 12-month period, from the 1st of October 2019 to 30th of September 2020, inclusive. An ongoing surveillance database capturing all presentations of nursing home resident’s ED visits within Tallaght University Hospital was utilized.

Clinical variables recorded included age, gender, vision, hearing, dementia and mild cognitive impairment. The patient’s mobility status and mobility aids were recorded. A presenting complaint of falls, and a history of recurrent falls (defined as two or more falls in the preceding 6 months) was also documented. Medications were included, taken from admission, via symphony or admission proformas and discharge letters. This included falls risk medications.

Barthels score was documented from the GEDI team, a Geriatric Emergency Department Interventional, team, if they were seen on admission. This team is available in the ED, Monday to Friday, 8 am to 5 pm, and comprises of a physiotherapist, speech and language therapist, occupational therapist, social worker and medical team. Time and date of presentation was recorded, along with referral source, via nursing home staff, or GP, and admission rates were also noted.

### Statistical analysis

Data were stored on an electronically security password protected Excel database accessible only by the chief investigators. A ‘fall’ was defined as a patient who presented with a fall or collapse or injury. Data were divided into two groups with two subtypes; these included patients who presented to the ED with (1) a fall with a falls history in the last 6 months, (2) a fall and no previous fall history, (3) presented to ED with a reason other than a fall and no falls history, (4) presented to ED with a reason other than a fall and had a falls history in the last six months.

Data were entered and analysed on SPSS 24.0 (SPSS Inc, USA) for statistical analysis. Descriptive statistics were used to present the study data as frequency and percentage. For normally distributed data, mean and standard deviations are presented. For skewed data median and interquartile range (IQR) were used. For comparing different groups (falls and non falls; gender, and referral sources) non-parametric data analysis was used. The Pearson chi-square test and the *t*-test were used to identify factors, which distinguished between the groups. The threshold for statistical significance was *P* < 0.05.

### Ethical approval

Ethics and Health Research Consent Declaration Committee approval was in place for longitudinal study of older people attending ED.

## Results

### Study population demographics

Data were analysed in two groups: ‘falls’, i.e. nursing home residents who presented to the ED with a history of falls, and ‘no falls’, i.e. nursing home residents who presented to the ED with a history of something other than a fall. Over the 52-week period there was 519 emergency visits by nursing home residents (see Table 1).

**Table 1.** Study Population Demographics.

					Count	Mean	Median	Percentile 25	Percentile 75	
Group	Falls	Gender	Female	Age	137	79.14	80.00	71.00	89.00	*P* = 0.165
			Male	Age	113	76.52	77.00	71.00	85.00	
		History of falls in past 6 months	Yes		205 (39.5%)					
			No		45 (8.7%)					
		Vision	Normal		160					*P* = 0.024
			Impaired		83					
		Hearing	Normal		215					*P* = 0.327
			Impaired		28					
		Dementia	No		119					*P* = 0.148
			Yes		124					
		MCI	No		225					*P* = 0.295
			Yes		20					
	No falls	Gender	Female	Age	131	74.03	75.00	65.00	85.00	
			Male	Age	138	75.07	76.00	67.00	82.00	
		History of falls in past 6 months	Yes		1 (0.2%)					
			No		268 (51.6%)					
		Vision	Normal		195					
			Impaired		65					
		Hearing	Normal		236					
			Impaired		23					
		Dementia	No		143					
			Yes		115					
		MCI	No		24					
			Yes		15					

#### Age and sex

The mean age for the falls group was 77.96 (±11.28), and for the no falls was 74.56 (±11.75) (*P* < 0.001 (CI 1.4-5.38)).

#### Dependency

There were different levels of dependency throughout the groups. A Barthel score was documented in 70.7% of cases (*n* = 367). The mean Barthel Index for patients who presented with a fall was 9.55 (±5.12) and 7.92 (±6.8) for patients who did not present with a fall (*P* < 0.001, CI 0.064-0.475).

### Medications and mobility aids

See Table 2.

**Table 2.** Medications and mobility aids.

		Group		Total	
		Falls	No falls		*P* value
Anticholinesterase inhibitor	No	198	220	418	0.112
	Yes	35	25	60	
Opiates	No	215	214	429	0.076
	Yes	18	31	49	
Benzodiazepines	No	152	166	318	0.560
	Yes	81	79	160	
Other psychotropic medication	No	41	51	92	0.349
	Yes	193	193	386	
Night sedation	No	170	180	350	0.900
	Yes	63	65	128	
Bone health	No	156	181	337	0.011
	Reviewed	53	33	86	
Mobility aids	No aid	60	60	120	<0.001
	Walking stick	19	9	28	
	Frame	83	41	124	
	Wheelchair	27	39	66	
	Hoist	27	73	100	

### Pre-hospital care and visit details

Twenty-six percent (26%, *n* = 134) of patients were referred by a general practitioner prior to transfer to hospital, while the rest presented prior to a review by a doctor (*n* = 375).

Over a third of ED visits (199/519, 38.34%) were within the normal working hours Monday to Friday. 35.6% of patients who presented with falls presented within working hours Monday to Friday (*n* = 89) in comparison to 40.89% of patients who did not present with falls (*n* = 110). 36.8% (*n* = 92) of patients who presented with falls presented in the evenings or overnight, and 27.6% (*n* = 69) presented at the weekend. Twenty-nine percent (29%) of patients who did not present with a fall presented in the evenings (*n* = 78), and 30.11% presented at weekend (*n* = 150). There was no statistical difference between the groups (*P* = 0.162). Patients were overall more likely to have been referred by a GP in hours, than overnight or at the weekend (*P* < 0.001) (see Table 3).

**Table 3.** Hours of Presentation.

Group			Referral source		Total
			Nursing home	Own GP	
Falls	In hours/OOH/weekend	In hours	61	26	87
		Out of hours	65	27	92
		Weekend	58	11	69
	Total		184	64	248
No falls	In hours/OOH/weekend	In hours	66	40	106
		Out of hours	57	18	75
		Weekend	66	12	78
	Total		189	70	259
Total	In hours/OOH/weekend	In hours	127	66	193
		Out of hours	122	45	167
		Weekend	124	23	147
	Total		373	134	507

### Clinical outcomes

See Table 4.

**Table 4.** Admission Rate.

		Group		Total	
		Falls	No falls		
Admitted (Y/N)	Admitted	137	179	316	*P* = 0.053
	Did not wait	0	3	3	
	Fracture clinic	7	7	14	
	Home	44	35	79	
	OPD	6	2	8	
	Other	0	1	1	
	Other hospital	8	6	14	
	Own GP	48	34	82	
	RIP	0	2	2	
Total		250	269	519	

#### Admission rate

66.67% (250/375) of the patient’s referred to the ED by the nursing home staff were admitted, whereas 46.29% (62/134) patients who were referred by their GP were admitted (*P* < 0.001). Subdivided, within the falls group, patients were more likely to be admitted if they were not reviewed by a GP prior to transfer (*P* < 0.001). The same was not true for the no falls group (*P* = 0.172).

#### Referral hours

There was no statistical difference within the falls and non falls groups, for rate of admission and hours of referral (*P* = 0.793, *P* = 0.411, respectively).

#### Length of stay

The average length of stay for these patients with a fall who were admitted was 10.4 (±17.95) days, in comparison to patients who did not present with a fall, with an average of 9.14 (± 8.74) days, (independent *t*-test: *P* = 0.410).

#### Injuries

Amongst documented admission/discharge diagnosis, the most common included were collapse (*n* = 58), head injury (*n* = 8), laceration (*n* = 5). Documented fractures included femur (*n* = 2), humerus (*n* = 1), ankle (*n* = 3), clavicle (*n* = 1), hand (*n* = 1), facial bones (*n* = 1).

## Discussion

This study finds that falls were an indication for almost half of all nursing home residents’ presentations to the ED. These presentations were associated with older age, vision impairment, the use of certain walking aids and a high Barthel score on admission. Current data suggest that women fall more than men, and tend to be more frail than man however gender differences in reporting may be a factor.^11^^,^^12^ This study found that females were more likely to present with falls, however it was not statistically significant (*P* = 0.165). Visual impairment and cognitive impairment also appeared to correlate with an increase in falls risk. There are few studies available associating these impairments with falls in nursing homes.

Visual impairment should not be overlooked as a risk factor for falls, as it is potentially modifiable. Poor vision can impair balance, and lead to an increased falls risk in the older population. The use of glasses can also incur a falls risk, as the perception of depth, sensitivity to contrast and ability to navigate obstacles can all be impaired.^13^ Hearing impairment is not typically associated with falls; however, it can lead to decreased auditory and spatial awareness. Previous studies have associated even a mild hearing loss of 25 dB, has been linked with a treble in reported falls in the preceding year.^14^ This study did not show that impaired hearing had a significant impact in risk of falls.

Dementia is known to be a risk factor for falls.^15^ This can be for many reasons and changes in the patient’s physiology; in insight, ability to recognize sensory input, ability to communicate, co-ordinate movement and interpret the environment.^16^ Patients are also less likely to retain new information and initiate tasks, when can lead to further immobility. Going forward, cognitive screening and staging, along with interventional programmes to target falls reduction in residents with dementia, may assist with preventing falls in the future.

This study verified that the use of a walking aid is a strong risk factor for falls amongst nursing home residents. Walking aids have been known to be associated with falls risks, however few studies have looking into the risk of falls in nursing homes with the various aid used by residents. Patients with falls were more likely to have a Zimmer frame than patients who presented without falls. Some studies have previously found the use of a walking aid to be a protective measure against future falls. However, the conclusion that fallers more often use walking aids than non-fallers, and recognition that walking aids are an individual risk factor for falls, specifically Zimmer frames, is in harmony with preceding research.^10^^,^^17^^,^^18^

There are many variations in the referral rates from GPs and many factors that influence the referral rate. These can include patient factors such as diagnosis and co-morbidities. Others include GP factors such as satisfaction with the specialty, importance of making/confirming a diagnosis and availability of facilities within their practice. Further external issues such as the presence of external referral and management guidelines, previous experience and the possibility of future litigation or complaint can also impact referral rates.

There is no consensus on the efficacy of osteoporosis medications in nursing home residents. This study demonstrated that falls patients were more likely to have their bone protection medications optimized. If fractures are sustained secondary to bone fragility, it is generally a signal that more fractures may occur. The assessment and investigations for osteopenia and osteoporosis, with lifestyle advice and treatments can reduce the re-fracture risk in many patients.^19^ This study is lacking on information regarding FRID medication doses, and the indication for starting medications. Medications such as antidepressants, benzodiazepines and antipsychotics have been reported to be a risk factor for falls, however the relationship was not statistically significant in this study.

Some residents in nursing homes do not get enough physical activity, and this can exacerbate the decline in gait and cognition, causing a reduction in muscle strength, walking and balance. Previous data have shown a positive impact of falls prevention programmes within nursing homes, by preventing deterioration and improving fall related outcomes in residents. The benefits of exercise programmes may improve cognitive function or slow down the decline of same in patients with dementia.^20^^,^^21^

### Limitations

Strengths of the study were the year long period, with a large population in the study. There were limitations to this study. As it is a retrospective cross-sectional study, the analysis cannot determine causality regarding what brought the nursing home residents to the ED. It relied on the accuracy of the documentation by nursing and medical staff in the ED. This may include under-reporting, misclassification, missing data and inconsistencies in data collection. There are potential errors due to missing data, miscoding and data linkage. This study was conducted on a single site; therefore, these findings may not represent care in other hospitals. This is having a significant impact on falls prevention programmes and may provide a better consensus as to why patients present in the first instance to the ED for falls. It is also noted that residents do not always wear glasses or hearing aids. It was not possible to determine if they were wearing them at the time of said fall.

## Conclusion

This study identifies many challenges in providing care to nursing home residents. As societies modernize, members begin to reduce the size of their families.^22^ This, coupled with emigration and an increase in women in workforce in recent decades, has and is continuing to lead to an increase in cohort of nursing home residents. All areas with involvement in the care of nursing home residents should have policies *in situ* for managing these patients and be actively involved in auditing their activity.

To decrease the falls risk going forward, one can ensure that patients under the care of a health professional or caregiver, should have once a year falls assessment if they have reported a recent fall.^23^ Medications should be optimized as deemed necessary, with a special look at bone protective measures as previously mentioned.

It is not the duty of a hospital to decide which admissions are potentially avoidable, due to the variance in nursing homes facilities, and individual factors justifying an emergency referral. These factors are possibly best advised by a multidisciplinary team with expertise in care of the elderly at hospital sites, who can engage with GPs, and nursing home managers within the catchment region.
